# Mechanism of Elian granules in the treatment of precancerous lesions of gastric cancer in rats through the MAPK signalling pathway based on network pharmacology

**DOI:** 10.1080/13880209.2021.2017980

**Published:** 2021-12-28

**Authors:** Zhirong Yi, Qingling Jia, Yili Lin, Yujiao Wang, Jun Cong, Zhijian Gu, Jianghong Ling, Gan Cai

**Affiliations:** a The First Affiliated Hospital of Guangxi Medical University, Guangxi Medical University, Nanning, People’s Republic of China; b Shuguang Hospital, Shanghai University of Traditional Chinese Medicine, Shanghai, People’s Republic of China

**Keywords:** Chinese medicine formula, molecular mechanism, bioinformation technology, biological network analysis, digestive disease

## Abstract

**Context:**

Elian Granules have been applied in the treatment of precancerous lesions of gastric cancer (PLGC) and achieved good results. However, its exact mechanism remains unclear.

**Objectives:**

To explore the mechanism of Elian granules in treating PLGC through the mitogen-activated protein kinase (MAPK) signalling pathway based on network pharmacology.

**Materials and methods:**

Through network pharmacological methods, the targets of the active component of Elian granules against PLGC were obtained. Subsequently, Specific Pathogen Free (SPF) male Sprague Dawley (SD) rats were randomly divided into normal, model, and Elian granule groups. The *N*-methyl-*N*′-nitro-*N*-nitrosoguanidine comprehensive method was used to establish the PLGC rat model. The model and Elian granule groups were given normal saline and Elian granule aqueous solution (3.24 g/kg/d) intragastric administration, respectively, for 24 weeks. The pathological changes in gastric tissues were observed by hematoxylin-eosin staining. The protein expression of p-JNK and p-p38 was verified by western blotting.

**Results:**

394 and 4,395 targets were identified in Elian granules and PLGC, respectively. The 190 common targets were mainly enriched in MAPK signalling pathways. The gastric mucosal epithelium was still intact, the glands were arranged regularly, and no goblet cells or apparent inflammatory cell infiltration were observed in the Elian granule group. The expression of p-JNK and p-p38 protein of the Elian granule group (0.83 ± 0.08; 1.18 ± 0.40) was significantly higher than the model group (0.27 ± 0.14; 0.63 ± 0.14) (*p* < 0.01; *p* < 0.05).

**Discussion and conclusions:**

Elian granules may play a critical role in the treatment of rat PLGC by up-regulating the expression of p-JNK and p-p38 proteins in the MAPK signalling pathway, thus providing a scientific basis for clinical application.

## Introduction

Precancerous lesions of gastric cancer (PLGC) refer to intestinal metaplasia and/or dysplasia based on gastric mucosal atrophy. Gastric cancer is the third leading cause of cancer deaths, and its formation goes through the process of chronic superficial gastritis → atrophic gastritis → intestinal metaplasia → dysplasia → gastric cancer (Correa et al. 1975). Atrophic gastritis with intestinal metaplasia and/or dysplasia is often called PLGC (Correa 2013; Bray et al. 2018; Rawla and Barsouk 2019). Therefore, effective prevention and treatment of PLGC are of great significance to reduce gastric cancer incidence and mortality. At present, Western medicine for the treatment of PLGC has not yet formed a unified, standardised, and effective treatment plan. Traditional Chinese Medicine has the effect of blocking or delaying malignant transformation, has low toxicity, few side effects (Hong and Mo 2018; Tian and Chen 2019), and is easily accepted by patients in the treatment of PLGC; therefore, it has attracted increasing attention.

Elian granules were created by Professor Cai Gan through decades of clinical experience. Elian granules have the function of clearing heat-toxin, promoting blood circulation, and invigorating the spleen. Elian granules are mainly prescribed for chronic atrophic gastritis (CAG) with intestinal metaplasia and dysplasia. The prescription has been used in the clinic for more than 30 years and has achieved good results (Cong et al. 2015; Gu et al. 2015). Animal experiments also show that Elian granules can effectively improve the atypia of gastric mucosal epithelial cells in an *N*-methyl-*N*′-nitro-*N*-nitrosoguanidine (MNNG)-induced carcinogenesis model in rats by blocking or even reversing the development of experimental precancerous lesions (Cai et al. 2000; Wang et al. 2001). However, the exact mechanism of action of Elian granules remains to be further studied.

**Figure 1.**
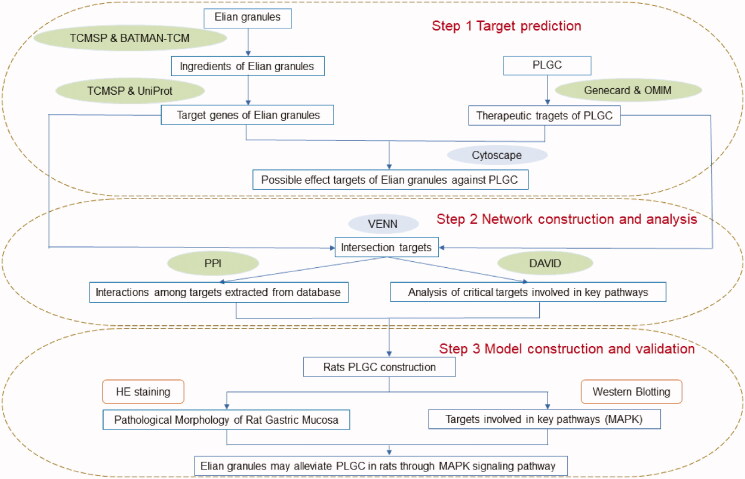
Technical route of the research based on network pharmacology and experimental validation. Abbreviations: TCMSP: Traditional Chinese Medicine Systems Pharmacology Database and Analysis Platform; BATMAN-TCM: A Bioinformatics Analysis Tool for Molecular Mechanism of Traditional Chinese Medicine; OMIM: Online Mendelian Inheritance in Man; PPI: protein-protein interaction; DAVID: the Database for Annotation, Visualisation and Integrated Discovery; PLGC: precancerous lesions of gastric cancer; MAPK: mitogen-activated protein kinase.

Network pharmacology is a new method covering traditional pharmacology, bioinformatics, chemical informatics, and network biology. Network pharmacology can systematically reveal the active components and potential mechanism of Traditional Chinese Medicine (Zhou 2015). Through network pharmacology, we predicted that Elian granules may treat PLGC by regulating the mitogen-activated protein kinase (MAPK) signalling pathway. A PLGC rat model was reproduced using the MNNG comprehensive method (Meng and Lei 1997; Yi et al. 2021). After intervention with Elian granules, the pathology of gastric tissue was assessed to verify the expression of p-JNK and p-p38, the two key protein markers in the MAPK signalling pathway, to confirm the role of Elian granules in preventing and treating PLGC through the MAPK pathway (Figure 1).

## Material and methods

### Collection, screening, and target prediction of the chemical composition of Elian granules

The single herbs Curcumae Rhizoma (*Curcuma phaeocaulis* VaL. [Zingiberaceae], Ezhu), Salviae Miltiorrhizae Radix et Rhizoma (*Salvia miltiorrhiza* Bge. [Labiatae], Danshen), Angelicae Sinensis Radix (*Angelica sinensis* (Oliv.) Diels [Umbelliferae], Danggui), Coptidis Rhizoma (*Coptis chinensis* Franch. [Ranunculaceae], Huanglian), Hedyotis Diffusa (*Hedyotis diffusa* Willd.[Oldenlandia diffusa (Willd.) Roxb.] [Rubiaceae], Baihuasheshecao), Codonopsis Radix (*Codonopsis pilosula* (Franch.) Nannf. [Campanulaceae], Dangshen), Atractylodis Macrocephalae Rhizoma (*Atractylodes macrocephala* Koidz. [Compositae], Baizhu), Glycyrrhizae Radix et Rhizoma (*Glycyrrhiza uralensis* Fisch. [Leguminosae], Gancao), Pinelliae Rhizoma (*Pinellia ternate* (Thunb.) Breit. [Araceae], Banxia), Citri Reticulatae Pericarpium (*Citrus reticulata* Blanco [Rutaceae], Chenpi), and Poria (*Poria cocos* (Schw.) Wolf [Polyporaceae], Fuling) were searched in the TCMSP database (https://tcmspw.com/tcmsp.php) (Ru et al. 2014; Ma et al. 2020). The active ingredients and corresponding target proteins of the above drugs were screened with oral bioavailability (OB) of ≥30% and drug-likeness (DL) at ≥0.18 as screening criteria. The chemical ingredients and corresponding target proteins of Taraxaci Herba (*Taraxacum mongolicum* Hand.-Mazz. [Compositae], Pugongying) were screened by setting Score ≥20 through A Bioinformatics Analysis Tool for Molecular Mechanism of Traditional Chinese Medicine (BATMAN-TCM) database (http://bionet.ncpsb.org.cn/batman-tcm/) (Liu et al. 2016). Finally, all the target names were converted into standard gene symbols using the UniProt database (https://www.uniprot.org/) (Magrane and UniProt 2011).

### PLGC target retrieval

“Precancerous Lesions of Gastric Cancer” was used as the keyword to search the Genecards (https://www.genecards.org/) and Online Mendelian Inheritance in Man (OMIM) (https://www.omim.org/) databases (Rebhan et al. 1997; Ada et al. 2005; Safran et al. 2010). False positives were removed to generate the final list of PLGC-related targets.

### Network construction and analysis

The potential Elian granules targets in the treatment of PLGC were then obtained by mapping the active ingredients-related targets of Elian granules with the PLGC-related targets. The relationship network of the “active component-action target” of Elian granules was constructed using Cytoscape 3.7.2 (https://cytoscape.org/index.html) (Shannon et al. 2003).

### Construction of protein-protein interaction network

The protein-protein interaction (PPI) network was constructed using the STRING database (https://string-db.org/) (Szklarczyk et al. 2011, 2019). The “Multiple Proteins” selection option, common intersection gene input, “Homo sapiens” protein species, and “medium confidence (0.400)” as the lowest interaction threshold were selected. The default settings were used for the remaining parameters. The generated PPI network data were then exported to construct the network map of Elian granules-related and PLGC-related targets in Cytoscape 3.7.2.

### Enrichment analysis

Gene ontology (GO) function analysis and Kyoto Encyclopaedia of Genes and Genomes (KEGG) pathway enrichment analysis of overlapping genes were carried out using the Database for Annotation, Visualisation and Integrated Discovery (DAVID) (https://david.ncifcrf.gov/) (Huang da et al. 2009). The significant functions and pathways were identified based on the critical value of *p* < 0.05, and the corresponding data were obtained for mapping.

### Experimental animals

Specific Pathogen Free (SPF) male Sprague Dawley (SD) rats aged 4–6 weeks and weighing 180 g ± 20 g were provided by the Experimental Animal Centre of Guangxi Medical University. The licence number for experimental animal production is SCXK (Gui) 2020-0003 and the licence number for experimental animal use is SYXK (Gui) 2020-0004. The animals were housed and fed in the SPF animal room of the Experimental Animal Centre of Guangxi Medical University (Ethics Number: 202006009).

### Main experimental reagents

MNNG (M0527) was purchased from Tixiai Chemical Industry Development Co., Ltd (Shanghai). Ranitidine hydrochloride capsules were from Guangdong Foshan Shouxin Pharmaceutical Co., Ltd., and 0.03% ranitidine hydrochloride SPF rat pellet feed was produced by Jiangsu Synergetic Pharmaceutical and Biological Engineering Co., Ltd. Elian granules (Z05101150) were provided by Shuguang Hospital affiliated to Shanghai University of Traditional Chinese Medicine. A bicinchoninic acid (BCA) kit (AR0146) was from Bausch Tech Bioengineering Co., Ltd. Rabbit anti-phospho-SAPK/JNK monoclonal antibody (mAb) (4668), rabbit anti-phospho-p38 MAPK mAb (4511), rabbit anti-GAPDH mAb (2118), and goat anti-rabbit IgG (5151) were purchased from Cell Signalling Technology (USA).

### Animal grouping, model production, and intervention

After 9 SPF male SD rats were fed a normal diet for one week, the rats were randomly divided into the normal, model, and Elian granules groups. The normal group was given SPF diet and clean drinking water. The model and Elian granules groups were used to produce the PLGC rat model via the MNNG comprehensive method. Briefly, (1) Rats were treated with the MNNG solution at 170 μg/mL via drinking. The MNNG water bottle was wrapped in tin foil to protect it from light. (2) Granular SPF rat feed containing 0.03% ranitidine hydrochloride was used for feeding. (3) The animals were fed for 2 days and then fasted for 1 day. (4) Intragastric administration of 2% sodium salicylate solution was performed in the afternoon of fasting at 10 mL/kg/d. In parallel, the model group was given normal saline intragastric administration (10 mL/kg/d) for 24 weeks. Intragastric administration of Elian granule aqueous solution (3.24 g/kg/d) was administered to the Elian granules group as an intervention for 24 weeks.

### Tissue sampling

After euthanization with 2% pentobarbital sodium (0.225 mL/kg) by intraperitoneal injection, the entire layer of gastric antrum tissue was excised from each animal for analysis. Part of the gastric tissue was fixed in 4% paraformaldehyde for routine paraffin sections and HE staining. The remaining tissue was frozen at −80 °C for western blotting (WB).

### Detection of gastric histopathology by HE staining

The paraffin section of the gastric antrum was stained with HE, and the pathological changes of gastric tissue were observed under a light microscope.

### Detection of p-JNK and p-p38 protein expression in rat gastric tissue by WB

Sodium dodecyl sulfate-polyacrylamide gel electrophoresis (SDS-PAGE) of the tissue lysates from each group was performed on 10% gel at 80 V for 30 min and then at 120 V for 60 min. After the protein was transferred to the PVDF membranes (Millipore, USA) at 300 mA for 90 min, the membranes were blocked with 5% bovine serum albumin (BSA) at room temperature with constant agitation for 60 min. The primary antibody solution (p-JNK, 1:500; p-p38, 1:500; GAPDH, 1:1000) was then added to each membrane and incubated overnight at 4 °C with constant shaking. The following day, the membranes were rinsed 3 times for 10 min each in 1X Tris Buffered Saline with Tween® 20 (TBST) and incubated with the corresponding species of infra-red-conjugated secondary antibody (1:10000) at room temperature for 60 min, in a shaker and protected from light. The membrane was then analysed using a two-colour infra-red fluorescence imaging system (LI-COR Odyssey, USA) and protein band integral optical density (Integrated Absorbance, or IA) calculated using the formula average optical density × area. The expression level of the target protein was normalised relative to GAPDH.

### Statistical analysis

Using SPSS21.0 software, a comparative analysis was performed using One-Way Analysis of Variance (ANOVA) to compare among multi-groups followed by the least significant difference (LSD) comparison between any two groups. The difference in the test level of bilateral *α* = 0.05 and *p* < 0.05 was considered statistically significant.

## Results

### Active ingredients and targets of Elian granules

Using the TCMSP and BATMAN-TCM databases, 254 active ingredients of Elian granules were obtained, including 4 active ingredients in Curcumae Rhizoma, 65 in Salviae Miltiorrhizae Radix et Rhizoma, 2 in Angelicae Sinensis Radix, 14 in Coptidis Rhizoma, 8 in Taraxaci Herba, 7 in Hedyotis Diffusa, 21 in Codonopsis Radix, 7 in Atractylodis Macrocephalae Rhizoma, 93 in Glycyrrhizae Radix et Rhizoma, 13 in Pinelliae Rhizoma, 5 in Citri Reticulatae Pericarpium, and 15 in Poria. A total of 394 targets of Elian granules were obtained after retrieval and weight removal.

### Potential targets of Elian granules in the treatment of PLGC

**Figure 2.**
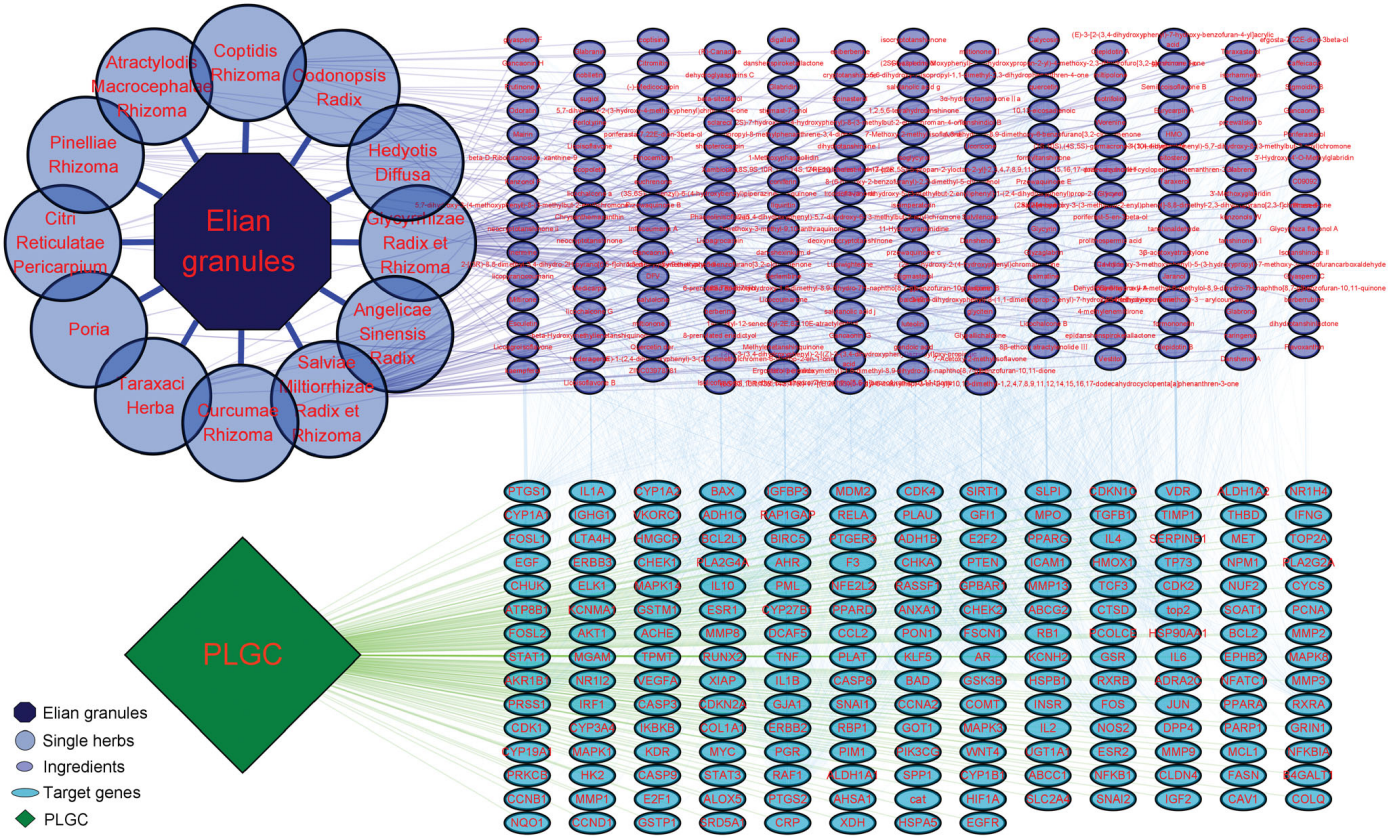
Elian granules, single herbs, ingredients, targets, and disease network construction using Cytoscape 3.7.2.

**Figure 3.**
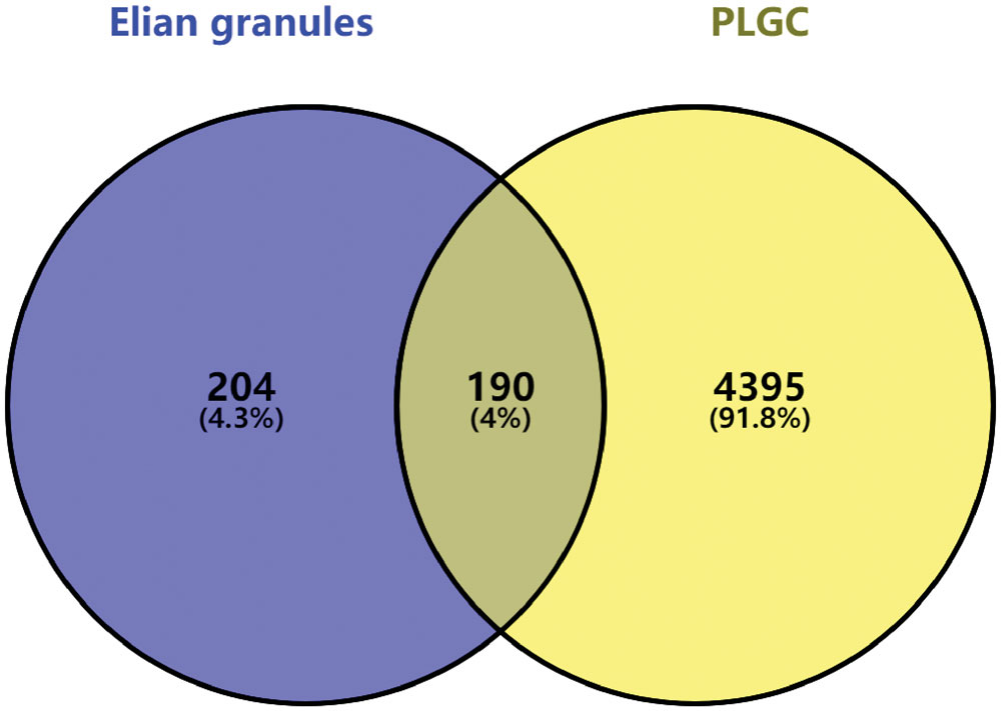
The intersection of Elian granules targets and disease targets using Venn diagram.

Via the Genecards and OMIM databases, 4,395 PLGC-related gene targets were identified. The “Elian granules-targets-PLGC” network was constructed by mapping with the targets of the main Elian granules ingredients. Of the 254 active ingredients of Elian granules, 190 showed a direct therapeutic effect on PLGC by acting on 190 targets (Figures 2 and 3).

### Key nodes of PPI network

**Figure 4.**
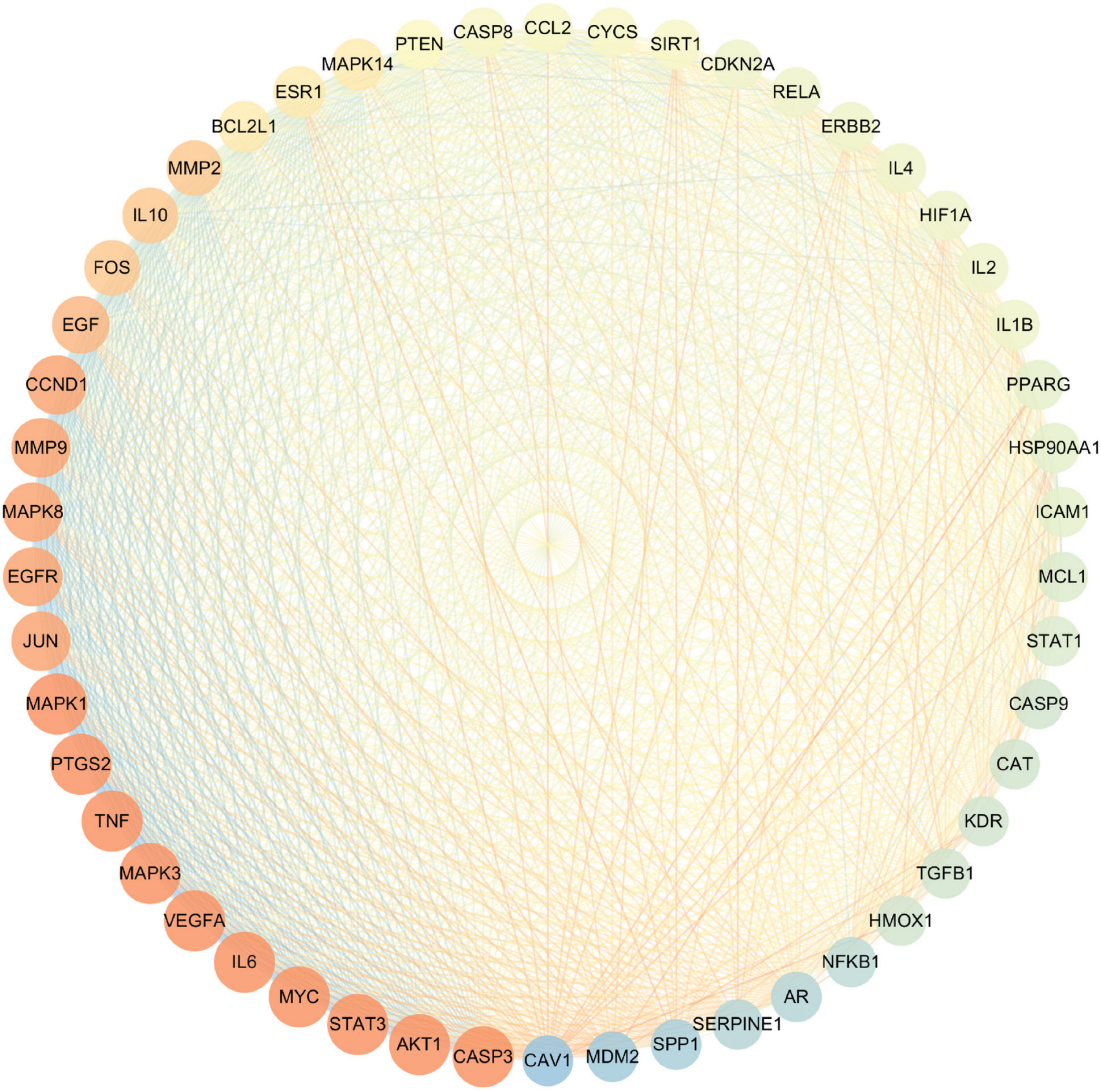
Protein-protein interaction network construction using Cytoscape 3.7.2. The size of the node represents the size of the degree. The more orange the node, the greater the degree, conversely, the bluer the colour, the smaller the degree.

To comprehensively explore the core pharmacological mechanism of Elian granules in the treatment of PLGC, we constructed a PPI network using the first 50 overlapping genes. Among them, the top three bases are AKT1, MAPK1 and MAPK3. The size of the gene node is related to the degree value. The larger the node, the more important the node is in the network (Figure 4).

### Enrichment analysis of the targets of Elian granules in the treatment of PLGC

**Figure 5.**
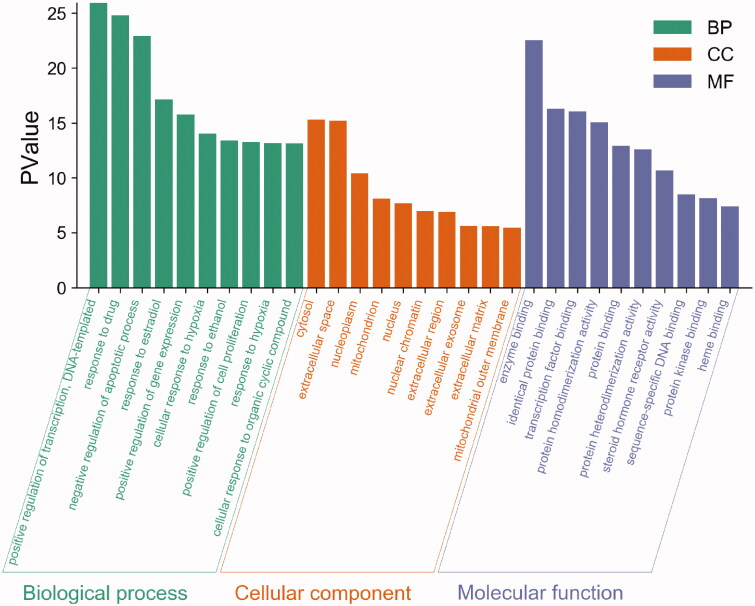
GO enrichment analysis of the 190 intersection targets by DAVID. The x-axis represents the top 10 significantly enriched terms in biological process, cellular component, and molecular function categories. The y-axis represents the *p*-value.

**Figure 6.**
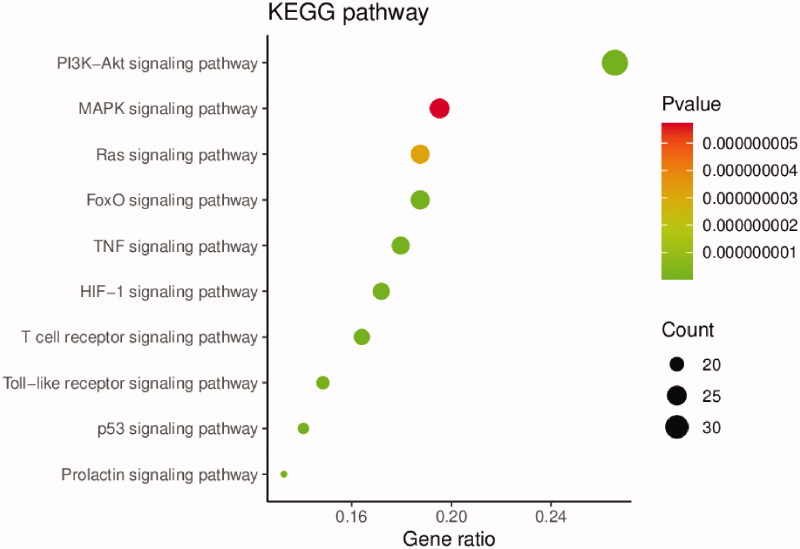
KEGG enrichment analysis of the 190 intersection targets by DAVID. The y-axis represents the top 10 significantly enriched pathways. The x-axis shows the ratio of enriched target genes to the background. The size of the dots displays the number of the target genes in the pathways, and their colour range reflects different *p*-values.

To further understand the pharmacological mechanism of Elian granules on PLGC, GO function, and KEGG pathway enrichment analyses of the 190 common targets were carried out using the DAVID database. The threshold was set at *P* < 0.05. We found the biological process function was mainly related to positive regulation of transcription DNA-templated, and drug response. The cellular component was primarily associated with the cytosol, extracellular space, and nucleoplasm. The molecular function was found mainly related to enzyme binding, identical protein binding, and transcription factor binding (Figure 5). KEGG analysis results showed that the treatment of PLGC with Elian granules was mainly related to autophagy and inflammation. The top 10 pathways were the PI3K-Akt, MAPK, FoxO, Ras, TNF, HIF-1, T cell receptor, Toll-like receptor, p53, and prolactin signalling pathways (Figure 6).

### Pathological changes in rat gastric tissue

**Figure 7.**
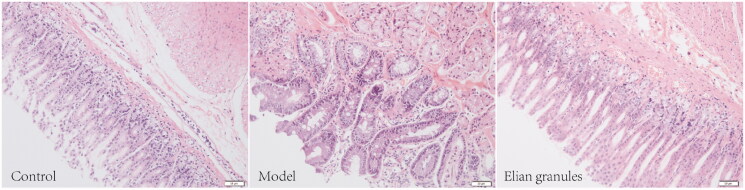
HE staining of gastric antrum tissue in Control, Model, and Elian granule groups (200×).

To observe the morphological changes of gastric mucosal epithelium in each group, HE staining was performed. In the normal group, the gastric mucosal epithelium was intact, the thickness was normal, and the glands were regularly arranged with an intact structure. No inflammatory cell infiltration was observed. In the model group, the thickness of the gastric mucosa was relatively normal. However, the gland arrangement was disordered, the shape was irregular, the glands decreased slightly, and goblet cells could be seen in the model group. In the Elian granules group, the gastric mucosal epithelium remained intact, the thickness was normal, the gland arrangement was approximately regular, the shape was relatively normal, and no goblet cells or obvious inflammatory cell infiltration was found (Figure 7).

### Expression of p-JNK and p-p38 protein in rat stomach tissue

**Figure 8.**
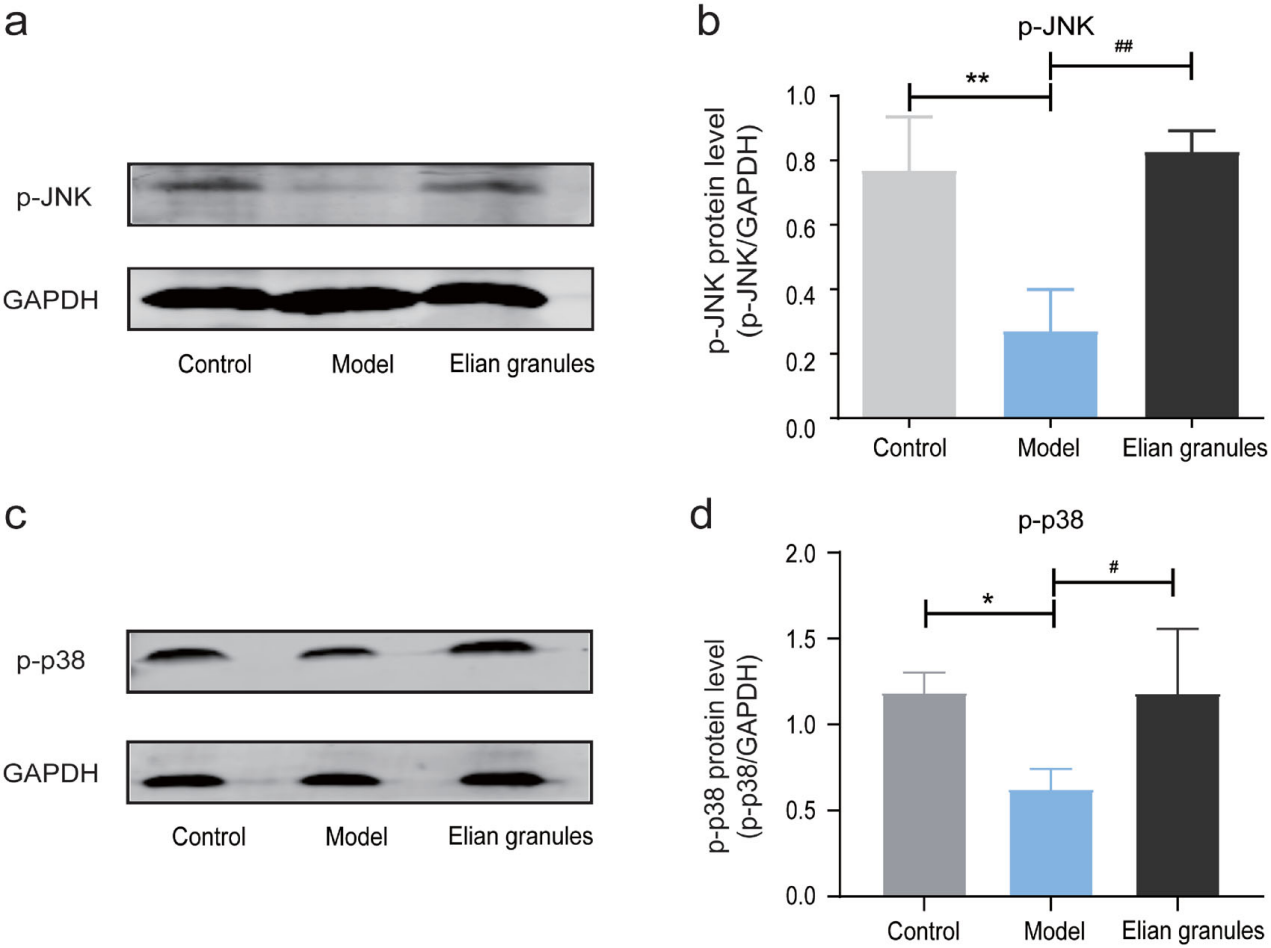
The expression of two key proteins in MAPK pathway using Western Blotting with GAPDH as the internal control. Figures (a–d) indicate the expression of p-JNK and p-p38 among Control, Model, Elian granule groups, respectively. Data were expressed as mean ± SD. Differences with *p <* 0.05 were considered statistically significant. *p < 0.05 and **p < 0.01, Model group vs. Control group; #*p* < 0.05 and ##*p* < 0.01, Elian granule group vs. Model group.

WB was carried out to detect the expression of p-JNK and p-p38 proteins in gastric mucosa. Compared with the normal group (0.77 ± 0.18; 1.19 ± 0.14), the expression of p-JNK and p-p38 protein in the gastric tissue of the model group (0.27 ± 0.14; 0.63 ± 0.14) was significantly decreased (*p* < 0.01; *p* < 0.05, respectively), whereas the expression of p-JNK and p-p38 protein in the gastric tissue of the Elian granules group (0.83 ± 0.08; 1.18 ± 0.40) was significantly higher than that of the model group (*p* < 0.01; *p* < 0.05, respectively) (Figure 8).

## Discussion

There is no record of the related disease name of PLGC in the ancient books of Traditional Chinese Medicine. According to its characteristics and main symptoms, PLGC may belong to the “Epigastric pain” and “Stomach ruffian” categories of Traditional Chinese Medicine. Professor Cai Gan, the first famous Traditional Chinese Medicine practitioner, thought that the pathogenesis of the disease is the weakness of the spleen and the stomach as the primary points, phlegm-dampness, heat toxin, qi stagnation, and blood stasis as the standard. Therefore, Cai Gan created Elian granules (formerly known as Le Wei Jian or Le Wei Fang). The entire prescription consists of Curcumae Rhizoma, Salviae Miltiorrhizae Radix et Rhizoma, Angelicae Sinensis Radix, Coptidis Rhizoma, Hedyotis Diffusa, Codonopsis Radix, Atractylodis Macrocephalae Rhizoma, Glycyrrhizae Radix et Rhizoma, Pinelliae Rhizoma, Citri Reticulatae Pericarpium, Poria, and Taraxaci Herba. The prescription uses Sijunzi decoction to replenish qi and invigorate the spleen. When combined with Pinelliae Rhizoma and Citri Reticulatae Pericarpium, it helps activate qi for lowering adverse qi and eliminate dampness and phlegm. Curcumae Rhizoma helps remove blood stasis and resolve static blood. Coptidis Rhizoma can clear fire and detoxify. However, when used at a lesser amount, it can also thicken the intestines and stomach. Hedyotis Diffusa and Taraxaci Herba help with clearing heat, detoxification, and carbuncle dispersing. Finally, Salviae Miltiorrhizae Radix et Rhizoma promotes blood circulation, relieves pain, cools blood, and eliminates carbuncle. All kinds of medicines are compatible with clearing heat and detoxification, activating blood circulation, and invigorating the spleen. Following the principle of combining deficiency and reality, rising and falling, strength and softness, and the combination of movement and stillness, more attention should be given to the adaptation of cold and warmth and the application of qi and blood at the same time. When Elian granules are applied clinically, the curative effects are found to be satisfactory (Cong et al. 2015; Gu et al. 2015). Animal experiments also showed that Elian granules could reverse the atypia of gastric mucosal epithelial cells and reduce DNA content and polyploidy in the MNNG rat model. This reversal effect may be achieved by inhibiting the expression of Bcl-2 and promoting the expression of Fas in the gastric mucosa of rats (Cai et al. 2000; Cui et al. 2000; Wang et al. 2001).

In this study, 394 potential targets of Elian granules were screened by network pharmacology. When intersected with 4,395 PLGC targets, 190 common targets were obtained. Enrichment analysis identified 674 biological processes, 89 molecular functions, 64 cellular components, and 57 signalling pathways associated with the 190 targets. We found that the 254 active Elian granules components mainly regulate JNK/MAPK8, p38/MAPK14, MAPK1, MAPK3, AKT1, TNF, EGF, and other related target proteins. They are also involved in regulating critical biological pathways, such as autophagy, inflammation, cell proliferation, and apoptosis, and may treat PLGC through the MAPK, PI3K-Akt, and other signalling pathways. MAPK is an intracellular signal transduction family of proteins, including JNK, p38, EPK1, and ERK2. Their activation and overexpression are related to tumorigenesis and development (Widegren et al. 2001). Previous studies have shown that the MAPK signalling pathway can effectively regulate the proliferation, migration, and invasion of gastric cancer cells (Sheng et al. 2020; Zhu et al. 2020; Wang et al. 2021). As one of the most important MAPK family pathways, JNK is mainly involved in stress response, apoptosis, and other physiological processes. JNK signal dysfunction is directly related to the occurrence and development of cancer, diabetes, and other diseases (Chu et al. 2019). The p38 signal transduction pathway is a mitogen-activated protein (MAP) kinase pathway, which plays an essential role in regulating inflammation, cell differentiation, cell growth, and cell death (Ono and Han 2000). In this study, using network pharmacology, our data indicate that Elian granules can regulate JNK/MAPK8 and p38/MAPK14, providing support for Elian granules in the treatment of PLGC through the MAPK signalling pathway.

We also employed the MNNG synthesis method to build a PLGC rat model tested with Elian granules intervention. The results of HE staining showed that, compared with the model group, the gastric mucosal epithelium of the Elian granules group had a more complete and regular glandular arrangement. The morphology was relatively normal, and no goblet cells were observed. WB results showed that p-JNK and p-p38 protein expression in the Elian granules group was significantly higher than that in the model group. These results confirmed that Elian granules may play a role in treating rat PLGC by up-regulating p-JNK and p-p38 protein expression in the MAPK signalling pathway. However, the mechanism of Elian granules in the treatment of PLGC still needs to be further studied.

Traditional Chinese medicine (TCM) is characterised by multi-component, multi-target, and multi-signal pathways in the treatment of tumours (Hong and Mo 2018). However, it is difficult to elucidate the exact mechanism of action of TCM. The innovative combination of network pharmacology and experimental validation has simplified this problem. In this study, the targets and signalling pathways of Elian granules were screened through the network pharmacology method and verified by animal experiments, which revealed its mechanism of action in treating PLGC and provided a scientific basis and ideas for future research.

However, this study still needs to be improved. The exact effective components of Elian granules were detected by mass spectrometry, different doses of Elian granules were set, and the median effective dose (ED_50_) and median lethal dose (LD_50_) indexes were discussed would make this study more scientific and rigorous. This will have important guiding significance for the clinical application of Elian granules and be the direction of further research.

## Conclusions

This study systematically identified the active ingredients and mechanisms of Elian granules in the treatment of PLGC through network pharmacology analysis and animal experimental verification. Namely, Elian granules play a critical role in the treatment of PLGC by up-regulating the expression of two key proteins, p-JNK and p-p38, in the MAPK signalling pathway, providing a preliminary foundation for the clinical application of Elian granules. However, since this study used SD rats as experimental subjects and the sample size was not large enough, further validation by multi-centre clinical trials is needed.

## References

[CIT0001] Ada H, Scott AF, Joanna A, Carol B, David V, McKusick VA. 2005. Online Mendelian Inheritance in Man (OMIM), a knowledgebase of human genes and genetic disorders. Nucleic Acids Res. 33(1):514–517. 10.1093/nar/gki033 PMC539987 15608251

[CIT0002] Bray F, Ferlay J, Soerjomataram I, Siegel RL, Torre LA, Jemal A. 2018. Global cancer statistics 2018: GLOBOCAN estimates of incidence and mortality worldwide for 36 cancers in 185 countries. CA Cancer J Clin. 68(6):394–424. 30207593 10.3322/caac.21492

[CIT0003] Cai G, Wang SP, Dou DB, Lin J. 2000. Effect of Lewei decoction on gastric mucosal epithelial cell dynamics in rats with gastric precancerous lesion. J New Chin Med. 32(2):35–37.

[CIT0004] Chu Q, Zhang Y, Zhong S, Gao F, Chen Y, Wang B, Zhang Z, Cai W, Li W, Zheng F, et al. 2019. *N*, *N*-Butyl haloperidol iodide ameliorates oxidative stress in mitochondria induced by hypoxia/reoxygenation through the mitochondrial c-Jun N-terminal kinase/Sab/Src/reactive oxygen species pathway in H9c2 cells. Oxid Med Cell Longev. 2019:7417561. 31205589 10.1155/2019/7417561 PMC6530120

[CIT0005] Cong J, Liao LJ, Zhu MP, Cai G, Zhang ZL. 2015. Clinical efficacy of Elian Granule in patients with chronic atrophic gastritis. J Anhui Tradit Chin Med Coll. 34(1):27–30.

[CIT0006] Correa P. 2013. Gastric cancer: overview. Gastroenterol Clin North Am. 42(2):211–217. 23639637 10.1016/j.gtc.2013.01.002 PMC3995345

[CIT0007] Correa P, Haenszel W, Cuello C, Tannenbaum S, Archer M. 1975. A model for gastric cancer epidemiology. Lancet. 306(7924):58–60. 10.1016/s0140-6736(75)90498-5 49653

[CIT0008] Cui RT, Cai G, Cheng Y, Wang SP, Yang QH, Tian T. 2000. Effect of Leweijian (LWJ) on experimentally induced dysplasia of gastric epithelial cell apoptosis and regulation gene. Chin J Integr Tradit West Med Gastrospleen. 8(2):67–69.

[CIT0009] Gu ZJ, Tang RY, Lin J, Li YM, Zhang ZL, Cai G. 2015. Elian Granules” for the treatment of chronic atrophic gastritis of spleen deficiency and heat stagnation pattern accompanied with intestinal metaplasia: a randomized controlled clinical trial. Shanghai J Tradit Chin Med. 49(4):40–43.

[CIT0010] Hong ZD, Mo ZX. 2018. 11 Kinds of signal pathways in anti-tumor mechanism of traditional Chinese medicine. Chin J Exp Med Formul. 24(21):205–218.

[CIT0011] Huang da W, Sherman BT, Lempicki RA. 2009. Systematic and integrative analysis of large gene lists using DAVID bioinformatics resources. Nat Protoc. 4(1):44–57. 19131956 10.1038/nprot.2008.211

[CIT0012] Liu Z, Guo F, Wang Y, Li C, Zhang X, Li H, Diao L, Gu J, Wang W, Li D, et al. 2016. BATMAN-TCM: a bioinformatics analysis tool for molecular mechanism of traditional Chinese medicine. Sci Rep. 6:21146. 26879404 10.1038/srep21146 PMC4754750

[CIT0013] Ma L, Tian GX, Geng H, Wang YJ, Zhang Y, Ma M, Lv J. 2020. Chinese medicine system pharmacology database TCMSP and its analytical application. Chin J Evidbas Cardiovascular Med. 12:1413–1416.

[CIT0014] Magrane M, UniProt C. 2011. UniProt knowledgebase: a hub of integrated protein data. Database (Oxford)). 2011:bar009. 21447597 10.1093/database/bar009 PMC3070428

[CIT0015] Meng ZX, Lei DN. 1997. Research progress on the mechanism of experimental adenocarcinoma induced by MNNG in animals. Foreign Med Sci. 17(3):136–139.

[CIT0016] Ono K, Han J. 2000. The p38 signal transduction pathway: activation and function. Cell Signal. 12(1):1–13. 10676842 10.1016/s0898-6568(99)00071-6

[CIT0017] Rawla P, Barsouk A. 2019. Epidemiology of gastric cancer: global trends, risk factors and prevention. Prz Gastroenterol. 14(1):26–38. 30944675 10.5114/pg.2018.80001 PMC6444111

[CIT0018] Rebhan M, Chalifa-Caspi V, Prilusky J, Lancet D. 1997. GeneCards: integrating information about genes, proteins and diseases. Trends Genet. 13(4):163–163. 9097728 10.1016/s0168-9525(97)01103-7

[CIT0019] Ru J, Li P, Wang J, Zhou W, Li B, Huang C, Li P, Guo Z, Tao W, Yang Y, et al. 2014. TCMSP: a database of systems pharmacology for drug discovery from herbal medicines. J Cheminform. 6(1):13. 24735618 10.1186/1758-2946-6-13 PMC4001360

[CIT0020] Safran M, Dalah I, Alexander J, Rosen N, Iny Stein T, Shmoish M, Nativ N, Bahir I, Doniger T, Krug H, et al. 2010. GeneCards version 3: the human gene integrator. Database. 2010:baq020. 20689021 10.1093/database/baq020 PMC2938269

[CIT0021] Shannon P, Markiel A, Ozier O, Baliga NS, Wang JT, Ramage D, Amin N, Schwikowski B, Ideker T. 2003. Cytoscape: a software environment for integrated models of biomolecular interaction networks. Genome Res. 13(11):2498–2504. 14597658 10.1101/gr.1239303 PMC403769

[CIT0022] Sheng YN, Luo YH, Liu SB, Xu WT, Zhang Y, Zhang T, Xue H, Zuo WB, Li YN, Wang CY, et al. 2020. Zeaxanthin induces apoptosis via ROS-regulated MAPK and AKT signaling pathway in human gastric cancer cells. Onco Targets Ther. 13:10995–11006. 33149614 10.2147/OTT.S272514 PMC7605660

[CIT0023] Szklarczyk D, Franceschini A, Kuhn M, Simonovic M, Roth A, Minguez P, Doerks T, Stark M, Muller J, Bork P, et al. 2011. The STRING database in 2011: functional interaction networks of proteins, globally integrated and scored. Nucleic Acids Res. 39(Database issue):D561–568. 21045058 10.1093/nar/gkq973 PMC3013807

[CIT0024] Szklarczyk D, Gable AL, Lyon D, Junge A, Wyder S, Huerta-Cepas J, Simonovic M, Doncheva NT, Morris JH, Bork P, et al. 2019. STRING v11: protein-protein association networks with increased coverage, supporting functional discovery in genome-wide experimental datasets. Nucleic Acids Res. 47(D1):D607–D613. 30476243 10.1093/nar/gky1131 PMC6323986

[CIT0025] Tian SD, Chen XY. 2019. Characteristics and advantages of Chinese medicine in the treatment of malignant tumors. Mod Chin Clin Med. 26(02):8–17.

[CIT0026] Wang PB, Chen Y, Ding GR, Du HW, Fan HY. 2021. Keratin 18 induces proliferation, migration, and invasion in gastric cancer via the MAPK signaling pathway. Clin Exp Pharmacol Physiol. 48(1):147–156. 10.1111/1440-1681.13401 32860257

[CIT0027] Wang SP, Cai G, Dou DB, Lin J. 2001. Effect of Leweijian on pathological atypism in rats with gastric precancerous lesions. Chin J Integr Tradit West Med Dig. 9(3):145–147.

[CIT0028] Widegren U, Ryder JW, Zierath JR. 2001. Mitogen-activated protein kinase signal transduction in skeletal muscle: effects of exercise and muscle contraction. Acta Physiol Scand. 172(3):227–238. 11472310 10.1046/j.1365-201x.2001.00855.x

[CIT0029] Yi JY, Yin J, Shi HL, Xu B, Chen Y, Fei XY. 2021. Research progress on development of animal models of chronic atrophic gastritis. Liaoning J Tradit Chin Med. 48(1):210–214.

[CIT0030] Zhou WX. 2015. Research progress and prospect of network pharmacology. Chin J Pharmacol Toxicol. 29(5):760–762.

[CIT0031] Zhu Q, Guo Y, Chen S, Fu D, Li Y, Li Z, Ni C. 2020. Irinotecan induces autophagy-dependent apoptosis and positively regulates ROS-related JNK- and p38-MAPK pathways in gastric cancer cells. Onco Targets Ther. 13:2807–2817. 32308415 10.2147/OTT.S240803 PMC7135144

